# Enhanced iturin a production in a two-compartment biofilm reactor by *Bacillus velezensis* ND

**DOI:** 10.3389/fbioe.2023.1102786

**Published:** 2023-01-19

**Authors:** Zhongmin Tang, Huili Zhang, Jie Xiong, Yang Li, Wei Luo

**Affiliations:** ^1^ College of Life Sciences, Shihezi University, Shihezi, Xinjiang, China; ^2^ The Key Laboratory of Industrial Biotechnology, Ministry of Education, School of Biotechnology, Jiangnan University, Wuxi, China

**Keywords:** biofilm reactor, iturin A, *Bacillus velezensis* ND, batch fermentation, step-wise temperature control

## Abstract

In this study, a two-compartment biofilm reactor was designed for iturin A production. The biofilm reactor consists of a stirred-tank fermentor containing exclusively suspended cells and a packing column where the biofilm is attached. Polyester fiber with sphere shape and rough surfaces was chosen as the carrier of biofilm in packing column. Batch, fed-batch, and repeated-batch fermentation using *Bacillus velezensis* ND in the biofilm reactor were studied. Compared to conventional suspended cell fermentations, the productivity of iturin A in batch and fed-batch biofilm fermentation were increased by 66.7% and 63.3%, respectively. Maximum itutin A concentration of 6.8 ± 0.1 g/L and productivity of 46.9 ± 0.2 mg/L/h were obtained in fed-batch biofilm fermentation. Repeated-batch fermentation showed high stability, with almost same profile as batch fermentation. After a step-wise temperature control strategy was introduced in the biofilm reactor, productivity of iturin A was increased by 131.9% compared to suspended cell reactor. This superior performance of biofilm reactor confirms that it has great potential in industrial production of iturin A.

## Highlights

• A two-compartment biofilm reactor was constructed to produce iturin A.
• The biofilm reactor exhibited high stability for repeated-batch fermentation.
• A maximum iturin A of 6.8 ± 0.1 g/L was produced in fed-batch fermentation.
• Step-wise temperature control fermentation improved the productivity of iturin A.

## Introduction

Iturin A is a cyclic lipopeptide containing seven residues of α-amino acids and one residue of a β-amino acid produced by the *Bacillus* strains. (Ongena and Jacques, 2008). Because of its broad-spectrum antifungal activity, iturin A is considered a powerful biopesticide in biological control of plant diseases. Iturin A has great potential as alternatives to conventional chemical pesticides in agriculture due to its low toxicity for human health and the environment (Hsieh et al., 2008; Arrebola et al., 2010; Raaijmakers et al., 2010; Velho et al., 2011; Meena and Kanwar, 2015). In addition to antimicrobial activity, insecticidal and anticarcinogenic properties of iturin A have been reported (Wan et al., 2021). Iturin A has gained more attention among the researchers for its wide application prospect in food, pharmaceutical, and agriculture.

To date, there have been many attempts to improve iturin A yield by bioprocess optimization and strain engineering (Rahman et al., 2007; Jin et al., 2015; Liang et al., 2020; Yue et al., 2021). Most researchers have employed conventional fermentation mode, submerged liquid fermentation or solid-state fermentation, for iturin A production. The production of iturin A reached 4.4 g/L (Mizumoto et al., 2007), 2.01 g/L (Xu et al., 2020) and 1.14 g/L (Chen et al., 2019) by optimizing the medium and fermentation process parameters. However, the low yield and the high production cost of iturin A still impede its large-scale production and utilization. It is worthwhile to develop novel cost-effective fermentation process to address the problem. Many *Bacillus* species can form rough biofilms at the air-liquid interface due to the aerotaxis of the cells. Therefore, these strains are potentially suitable for biofilm based bioprocess. Rahman et al., 2007 studied the production of iturin A by *Bacillus subtilis* 168 recombinant strain which was cultivated in beakers and multidish plates for biofilm formation. Compared with submerged fermentation, biofilm fermentation enhances the amount of production of iturin A and the final yield of iturin A was nearly doubled. This result revealed the potential of producing iturin A by biofilm fermentation. To date, however, there are not works on iturin A production in biofilm reactor.

Biofilm reactor allow microorganisms to self-immobilized and form a thick cell layer known as “biofilm” by providing suitable support materials (carrier). Biofilms have complex structure and provide the benefit of a stable environment for the enclosed microbes. These embedded cells exhibit different growth and bioactivity such as production of biofilm specific metabolites compared with suspended cells (Cheng et al., 2010). Other advantages of biofilm include the strong resistance of the attached biomass to toxic compound, high biomass density, as well as stability. Thereby, biofilms have great potential as industrial workhorses for the production of valued products and biofilm reactor employed in the biofilm fermentation has attracted the interest of researchers (Rosche et al., 2009; Ercan and Demirci, 2013). A number of products, such as bioethanol, organic acids, enzymes, antibiotics, vitamin, and polysaccharides, have been targeted (Khiyami et al., 2006; Demirci et al., 2007; Izmirlioglu and Demirci, 2016). In particular, several studies have investigated the production of surfactin and fengycin, the two other types of lipopeptides, in different biofilm bioreactors (Coutte et al., 2010; Chtioui et al., 2012; Chtioui et al., 2014; Brück et al., 2020). Their results showed the efficiency of the biofilm bioreactor for lipopeptides production since they provide process stability through cell immobilization and avoid foam formation. Other studies also shown that the lipopeptide productivity could be increased through cell immobilization (Brück et al., 2019). Biofilm reactors have been considered as promising systems for lipopeptides production. Thus, our effort was paid to explore the possibility of producing iturin A by biofilm reactor.

For an improved production in biofilm reactors, reactor design and the support materials are needed to be improved. In this study, a novel two-compartment biofilm reactor was designed for iturin A production. In order to increase the attachment between microorganisms and carrier, several different types of carrier were tested and the most appropriate one was selected for biofilm reactor construction. Batch, fed-batch and repeated-batch fermentation were studied to achieve a high iturin A concentration and high reactor productivity. Additionally, a step-wise temperature control strategy was introduced to further enhance the production of iturin A. The advantages of biofilm fermentation in the biofilm reactor, as compared to suspended cell fermentation, were also discussed in this article.

## Materials and methods

### Microorganism and growth conditions

*Bacillus velezensis* ND (CCTCC M 2020983, China Center for Type Culture Collection, Wuhan, China), which was isolated from a soil sample (Zhao et al., 2021), was used in this study. Strain was routinely cultured overnight in Luria-Bertani (LB) agar (15 g/L agar) plates at 37°C. The inocula were scraped and transferred into 100 mL of seed medium (pH 7.0) containing 10 g/L tryptone, 5 g/L yeast extract, and 10 g/L NaCl and cultivated at 28°C, 200 rpm for 18–20 h. Then the resulted seeds (10%, v/v) were inoculated into 50 mL of fermentation medium containing 120 g/L Soybean meal powder, 16 g/L yeast extract, 1 g/L monosodium glutamate, 88.3 g/L glycerol, 0.5 g/L MgSO_4_, 7H_2_O, 1 g/L KH_2_PO_4_, 0.15 mg/L FeSO_4_, 7H_2_O, 5 mg/L MnSO_4_·H_2_O, and 0.16 mg/L CuSO_4_.5H_2_O, and adjust the pH to 8.0 using 1 mol/L NaOH solution. The broth was incubated at 28°C in a rotary shaker with 200 rpm speed for 5 days for shake flasks fermentation.

### Selection of carrier for biofilm reactors

Six different porous carriers were tested, namely P (polyester fiber material), C (porous ceramic material), Q (Ceramic material), k1, k2, and k3 (polyethylene material). The particle size of carriers P, C, Q, k1, k2, and k3 was 4.0, 3.0, 2.6, 1.0, 1.5, 2.0 cm, respectively. The specific surface area was 3,000, 1800, 1,400, 600, 660, 750 m^2^/m^3^, respectively. Carrier (approximately 60% culture medium volume) was added into shaking flask fermentation to evaluate its effects on the cell weight and iturin A yield. These 250 mL flasks containing 50 mL of the fermentation medium were incubated at 28 °C with shaking at 200 rpm for 5 days.

### Two-compartment biofilm reactor construction and operation

**FIGURE 1:**
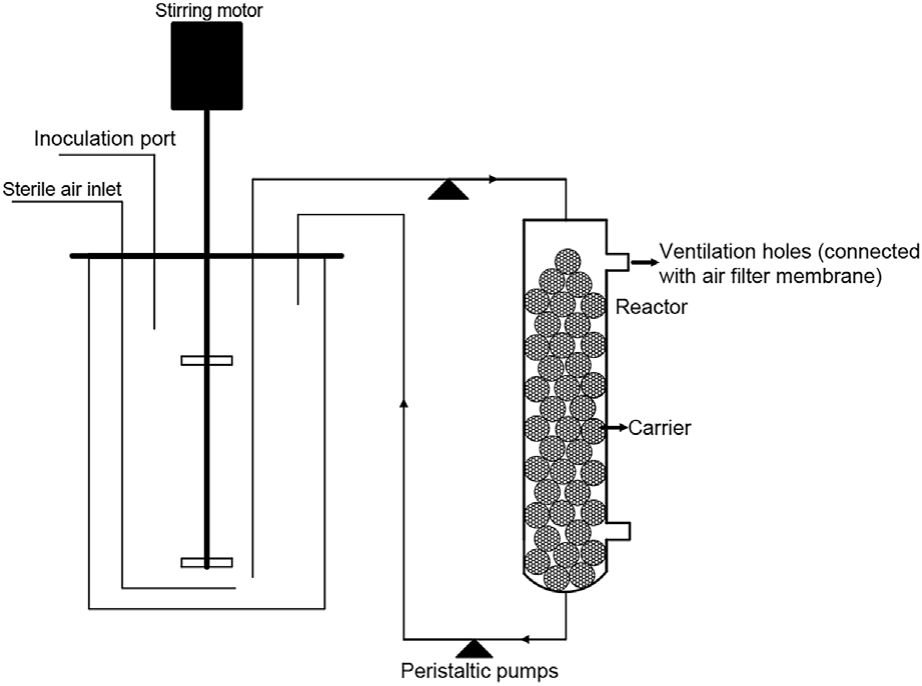
Schematic diagram of the two-compartment biofilm reactor.

A schematic diagram of the biofilm reactor is given in Figure 1. The system consists of two parts: (i) a 3 L stirred-tank fermentor (Baoxing Co., China) and (ii) a glass column reactor (with a diameter of 60mm and a height of 340mm) packed with carriers for biofilm formation. The glass column reactor was connected to the stirred-tank fermentor through a recirculation line. The entire reactor system contained 1 L of the medium and the liquid medium was recirculated continuously between these two parts with a flow rate of 18 mL/min *via* peristaltic pump. Unless otherwise noted, the stirred-tank fermentor was controlled at 28°C with stirring at 500 rpm and aeration at 3 vvm (air volume/culture volume/min). During the fermentation process, the stirred-tank reactor offers aerobic growth conditions for suspended cells and ensures a mixing of the medium. On the other hand, the broth trickles from the top of the glass column and flow down over the surface of carriers where the biofilm is attached. Then, the broth collected at the bottom of the reactor flows into the stirred-tank reactor. To control foam production, 20 mL polyether defoamer (Xinjiang Fufeng Biotechnologies Co., Ltd. China.) was added to the culture medium before fermentation. When the defoaming electrode detects foam generation in the stirred-tank fermentor during fermentation, defoamer will be automatically fed into fermentor.

### Batch and fed-batch fermentations in biofilm reactor

In batch fermentation, the reactor containing 1 L of medium was inoculated with seed culture of *Bacillus velezensis* ND (10%, v/v), and then the fermentation broth was circulated to the connected column reactor (4/5 volume of the reactor is filled with fiber ball carrier) by peristaltic pump for 8 days. Liquid samples were taken from the stirred-tank fermentor at regular intervals for the determination of suspended cell density, residual substrate and iturin A concentration. After each sampling procedure, sterile water was added to the fermentor to restore the fermentation volume to 1 L (High stirring rates and aeration can accelerate water evaporation in the fermentation broth, which will reduce the volume of the broth and cause viscosity). Fed-batch fermentation were carried out by adding steriled glycerol (carbon source) to the fermentor. The glycerol (18 g) was supplemented (one-time addition) at 48 h and 96 h respectively, at which point glycerol was less than 28 g/L.

### Batch and fed-batch fermentations in suspended cell reactor

For comparison, batch and fed-batch fermentations in the suspended cell bioreactor were carried out. Specifically, a 3 L stirred-tank fermentor containing 1 L of medium was inoculated with seed culture of *Bacillus velezensis* ND (10%, v/v) and fermented at 28°C. The aeration rate was controlled at 3 vvm and the stirring speed was 500 rpm. Fed-batch fermentation were carried out by adding steriled glycerol (carbon source) to the fermentor. The glycerol (18 g) was supplemented (one-time addition) at 96 h and 144 h respectively, at which point glycerol was less than 28 g/L. Biomass, yield and productivity of iturin A, carbon source consumption was compared between suspended cell and biofilm reactor.

### Repeated-batch fermentation in biofilm reactor

The repeated-batch fermentation in biofilm reactor was operated by replacing the fermented broth with 1 L of fresh medium and the culture immobilized on the carrier was used as the inocula for the next batch. Other fermentation conditions were the same as described in batch fermentation in biofilm reactor. Three repeated batch fermentation were carried out to assess the stability of iturin A production by the biofilm reactor.

### Batch fermentation in biofilm reactor with a step-wise temperature control strategy

The operating procedures for this method were as follows: fermentation temperature was set at 37°C from 0–24 h, 34°C from 24–36 h, 31°C from 36–48 h, and 28°C after 48 h. This strategy was used during batch fermentations in biofilm reactor. The fermentation conditions except temperature were the same as described in batch fermentation in biofilm reactor.

For comparison, batch fermentations in the suspended cell bioreactor with step-wise temperature control strategy were carried out.

### Analytical methods

For the determination of cell concentration in broth, 1 mL of fermentation broth mixed with 9 mL of sterile distilled water, and shaken by a vortex. Then, the mixture was serially diluted and spread onto LB-agar plates. After 12 h of incubation at 37°C, cell concentration was determined as colony-forming units per milliliter (CFU/mL).

For the determination of cell dry weight in broth, 10 mL sample was centrifuged at 5,000 rpm for 10 min. The cell were washed twice with 50 mL distilled water and dried to constant weight at 80°C.

For the determination of cell dry weight in biofilm, the carrier was taken out from the reactor to the gauze at the end of fermentation. The biofilm was stripped from the carrier with tweezers, then the biofilm with medium was washed with PBS buffer, and was dried to constant weight at 80°C.

The concentration of glycerol was determined with a Glycerol Assay Kit (APPLYGEN Co., China). The fermentation broth was centrifuged at 5,000 rpm for 10 min, and resulting supernatant was diluted 1,000 times. 5 μl of diluent was mixed with 195 ul detection buffer of the kit. After the mixture had been incubated at 37°C for 15 min, the glycerol concentration was determined by microplate reader at 550 nm.

For the determination of the concentration of iturin A, 0.3 mL of fermentation broth was added to 0.9 mL of methanol and the mixture was vigorously shaken using a vortex mixer for 10 min. The extract was centrifuged at 12,000 rpm for 15 min, and the supernatant was used for iturin A determination. The iturin A concentration was measured on an Agilent RP-HPLC (Agilent Technologies, United States), which equipped with Agilent Lichrospher C18 column (Agilent ZORBAX Eclipse XDB-C18 column, United States). The mobile phase was10 mM ammonium acetate/acetonitrile = (65:35, v/v), and flow rate was 1.0 mL/min. The injection volume was 20 μl and detection wavelength was 210 nm, and concentration of iturin A was calculated by the standard curve made by standard iturin A (purity 95%, Sigma-Aldrich United States).

For the determination of iturin A adsorbed on carrier, the carrier was taken out from the reactor at the end of fermentation and biofilm was rinsed thoroughly with 1,000 mL distilled water. Then, the mixture of biofilm and water was vigorously vortexed for 30 min to facilitate the disintegration of bacterial clumps and was used to extracted iturin A. The iturin A extraction and assay were performed as described above.

All assays were performed at least in triplicate, and data represent the mean of three or more experiments. All observations were repeated and the average values were obtained and demonstrated with standard errors of the repetitions as error bars. Using Origin 2018 ANOVA, any difference with *p* < 0.05 was considered significant.

## Results

### Selection of suitable carriers

**FIGURE 2:**
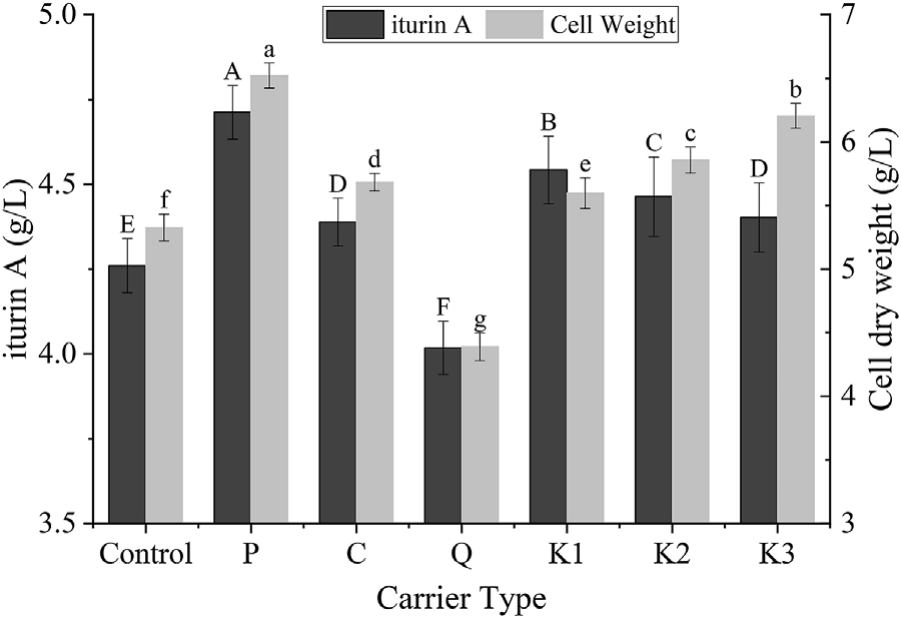
Effects of different carriers on the cell growth and the production of iturin A in *B. velezensis* ND in the shake-flask scale. Different letters meant there was significant difference among groups (*p* < 0.05).

Six different types of carrier were tested for their effects on the production of iturin A and cell growth so as to find a suitable carrier. As shown in Figure 2, P, C, k1, k2, and k3 demonstrated a significant promotion effect on cell growth and iturin A production (*p* < 0.05). This may be attributed to two factors: i) the carrier vibrated in a shake flask, promoting the oxygen and nutrient transfers in broth. ii) Biofilm formed gradually at the surface of these carriers, resulting in a higher amount of biomass. Unlike these carriers, Q appeared to have an inhibitory effect upon cell growth and biofilm was not observed at the surface. The reason of this unexpected observation was still unclear. It was probably due to unknown component of carrier Q which was made of natural raw materials. On the other hand, carrier P yielded the highest iturin A concentration (4.7 ± 0.1 g/L) and biomass (6.5 ± 0.1 g/L). These values were 10.6% and 22.3% greater than the control group (4.3 ± 0.1 and 5.3 ± 0.1 g/L, respectively). The better performance of P could be attributed to its larger specific area resulting from structural differences among the carriers. For these reasons, carrier P was selected as the most suitable packing for cell immobilization in biofilm reactor system and was used in the following experiments.

### Batch and fed-batch fermentations for iturin A production in the biofilm reactor

**FIGURE 3:**
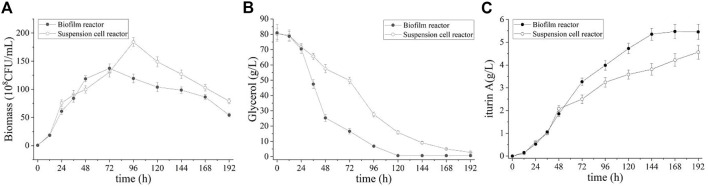
Time course of biomass **(A)**, residual glycerol **(B)** and iturin A production **(C)** during the batch fermentation in suspension cell reactor and biofilm reactor.

Batch fermentation was first performed to investigate the effectiveness of iturin A production by using the biofilm reactor. For comparison, batch and fed-batch fermentations in the suspended cell bioreactor were also carried out. Figure 3 shows the time courses of the biomass, residual glycerol and the concentration of iturin A in biofilm reactor and suspended cell reactor. As showing in Figure 3A, cell started growing rapidly both in suspended cell reactor and biofilm reactor during culture (0–72 h), and the maximum growth rate is almost the same. At 96 h of culture, suspended cell reactor achieved a highest biomass (184.0 ± 7.9 × 10^8^ CFU/mL) which is higher than biofilm reactor (137.3 ± 2.9 × 10^8^ CFU/mL). However, it should be noted that the biomass only represented growth in the broth and not in the biofilm. Actually, biofilm formed gradually in biofilm reactor and the total amount of biomass in broth and biofilm is higher than that in suspended cell reactor (Table 1). In both reactors, there was a rapid decrease in cell density after the culture reached its maximum concentration value, however, the iturin A production continued. This result indicated that iturin A is a non-growth-associated product. In biofilm reactor, faster glycerol consumption was observed and iturin A concentration reached maximum levels at the same time as the depletion of glycerol (Figure 3B). Figure 3C shows that iturin A concentration reached its maximum at 5.4 ± 0.1 g/L after 144 h in biofilm reactor and 4.6 ± 0.1 g/L after 192 h in suspended cell reactor. This implies that biofilm fermentation increases the yield by 17.3% and the productivity by 56.4% compared with suspended cell fermentation. In addition, 355.1 ± 31.7 mg iturin A was obtained from the biofilm on the carrier so that the total amount of iturin A in biofilm reactor was 5.7 ± 0.1 g/L which increased by 25.2% compared with the suspended cell fermentation.

**TABLE 1:** Comparison of batch and fed-batch fermentation of *Bacillus velezensis* ND in suspended cell reactor and biofilm reactor. Productivity was calculated as total iturin A produced (mg/L) in a given time (h). DCW: Cell dry weight; Y_X/S_:Yield of biomass per glycerol; Y_P/S_:Yield of iturin A per carbon source; Y_P/X_:Yield of iturin A per biomass; μ_max_ (in broth): Maximum specific growth rate.

Fermentation mode	Suspended cell reactor			Biofilm reactor		
	Batch	Fed-batch	Batch (step-wise temperature control)	Batch	Fed-batch	Batch (step-wise temperature control)
Time (h)	192	192	192	144	144	120
DCW (in broth) (g/L)	6.9 ± 0.2	7.4 ± 0.1	7.4 ± 0.2	6.2 ± 0.1	7.1 ± 0.1	7.0 ± 0.1
DCW (in biofilm) (g)	—	—	—	4.0	4.5	4.5
Iturin A (in broth) (g/L)	4.6 ± 0.1	5.5 ± 0.1	4.0 ± 0.1	5.4 ± 0.1	6.4 ± 0.1	6.2 ± 0.1
Iturin A (in biofilm) (mg)	—	—	—	355.1 ± 31.7	387.3 ± 22.1	379.2 ± 31.6
Total iturin A (g/L)	4.6	5.5	4.0	5.7	6.8	6.6
Productivity (mg/L/h)	23.8 ± 0.3	28.8 ± 0.3	20.9 ± 0.1	39.7 ± 0.2	46.9 ± 0.2	55.1 ± 0.3
Y_X/S_ (mg/g)	77.8	57.8	83.7	115.5	90.2	129.6
Y_P/S_ (mg/g)	51.6	43.0	45.4	64.7	52.7	74.8
Y_P/X_ (mg/g)	663.8	744.9	542.6	559.8	584.3	577.8
μ_max_ (/h)	0.29	0.30	0.36	0.28	0.30	0.40

**FIGURE 4:**
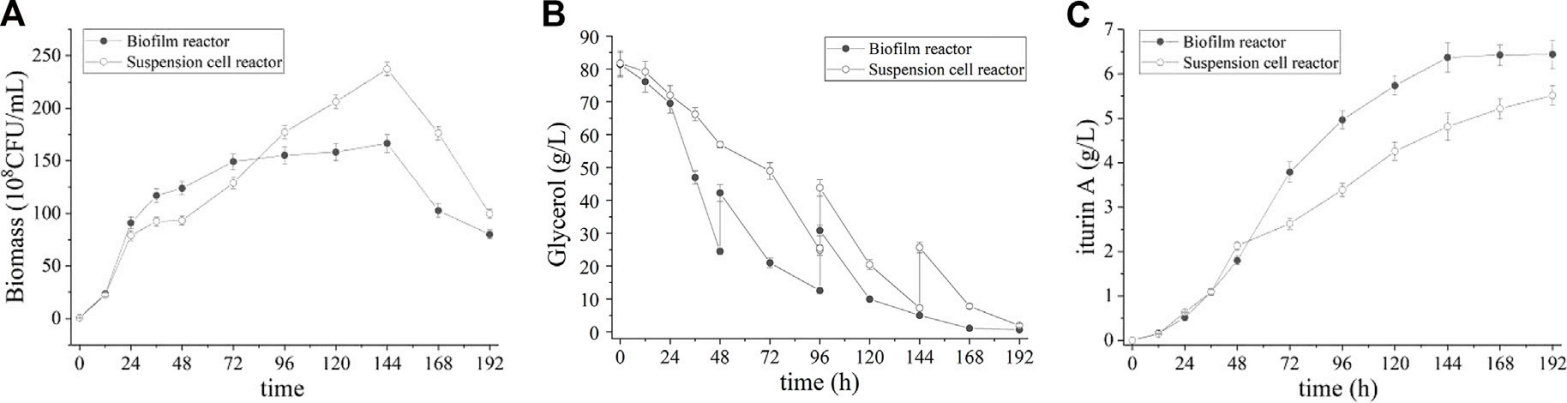
Time course of biomass **(A)**, residual glycerol **(B)** and iturin A production **(C)** during the fed-batch fermentation in suspension cell reactor and biofilm reactor.

The evidence of substrate limitation occurred in batch fermentation and substrate inhibition present at high glycerol level (data not shown) indicated the appropriateness of fed-batch fermentation. Figure 4 shows the time course of fed-batch fermentation in biofilm reactor and suspended cell reactor. The glycerol was supplemented in biofilm reactor at 48 h and 96 h respectively, at which point glycerol was less than 28 g/L. For suspended cell reactor, the glycerol comsuption is slower and glycerol was supplemented at 96 h and 144 h respectively. As showing in Figure 4A, the feeding of glycerol prolonged the cell growth phase and achieved highest biomass at 144 h in both reactor. Accordingly, a higher cell yield than batch process in both biofilm and suspended cell reactor (166.7 ± 4.7×10^8^ and 237.3 ± 5.8 × 10^8^ CFU/mL, respectively) was achieved. As shown in Figure 4C, iturin A reached it maximum at 6.8 ± 0.1 g/L after 144 h in biofilm reactor while 5.5 ± 0.1 g/L after 192 h in suspended cell reactor. Compared with batch process, the yield of iturin A is further improved.

### Repeated-batch fermentation in the biofilm reactor

**FIGURE 5:**
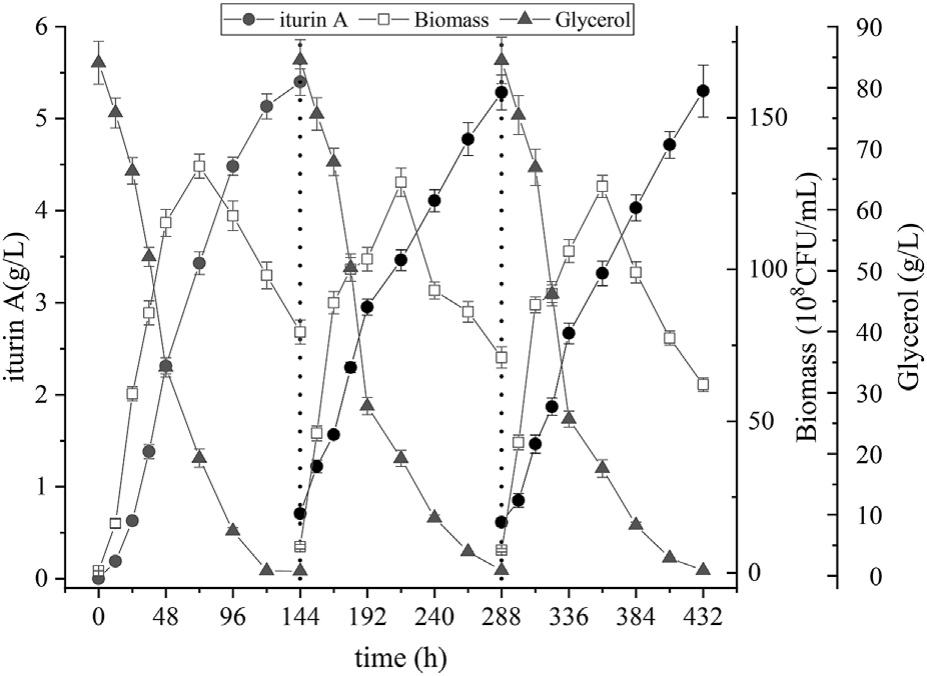
Time course of biomass, residual glycerol, and iturin A production during the repeated-batch fermentation in biofilm reactor.

Three cycles of repeated-batch fermentation were carried out in order to validate the stability of biofilm and long-term performance of the biofilm reactor. As shown in Figure 5, the profile of each fermentation cycle is nearly the same. Specific data of three cycles of repeated-batch fermentation are shown in Table 2. The yield of iturin A in three batches was 5.4 g/L, 5.3 g/L and 5.3 g/L, respectively, and the cells dry weight at the end of each batch increased gradually from 6.3 g/L to 6.9 g/L. Also, the trend of cell growth, substrate consumption and iturin A production in repeated-batch fermentation were nearly the same as single batch fermentation in biofilm reactor. These results indicated that the biofilm on the carrier could maintain their long-term stability, providing the seeds for the next batch. It should be noted that the repeated-batch process omitted inoculation requirements between fermentation cycles and saved costs for seed culture, but it did not showed a shorter period of fermentation and a higher productivity than single batch fermentation. However, long-term stability of biofilm in carrier implied a possibility of continuous fermentation process and the yield of iturin A could be further improved through continuous fermentation.

**TABLE 2:** Comparison of parameters in each cycles.

Repeated-batch cycles	Cell dry weight (g/L)	Iturin A (g/L)	Iturin A productivity (mg/L/h)
1	6.3 ± 0.1	5.4 ± 0.1	37.4 ± 0.2
2	6.6 ± 0.2	5.3 ± 0.1	36.7 ± 0.2
3	6.9 ± 0.1	5.3 ± 0.1	36.8 ± 0.1

### Batch fermentations in the biofilm reactor with a step-wise temperature control strategy

**FIGURE 6:**
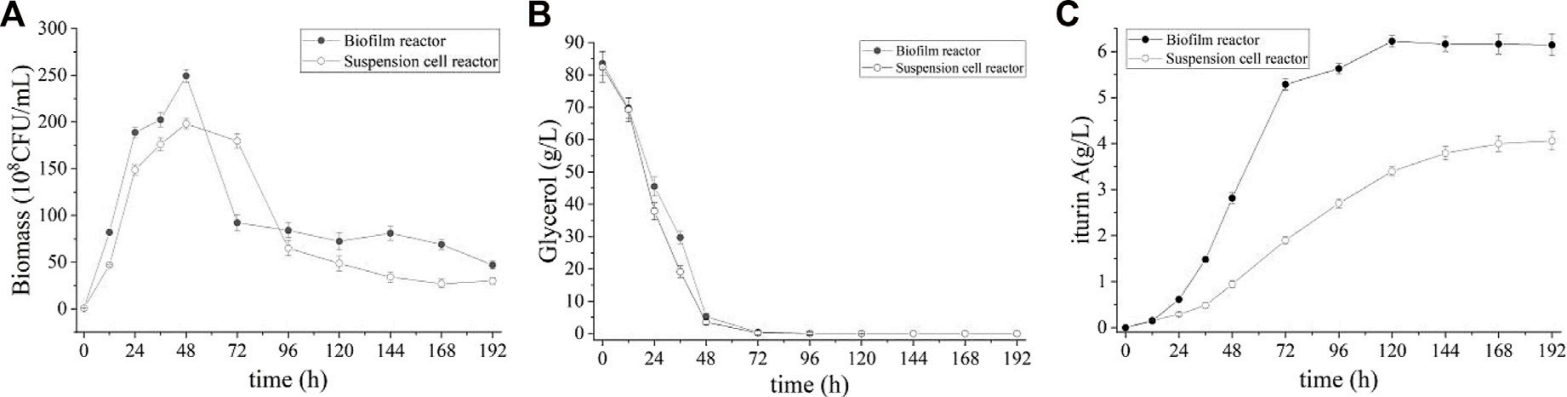
Time course of biomass **(A)**, residual glycerol **(B)**, and iturin A production **(C)** during the batch fermentation in suspension cell reactor and biofilm reactor with a step-wise temperature control strategy.

According to our previous study of this strain, the optimal temperature for good cell growth is 37 °C while the optimal temperature for iturin A production is 28°C (data not shown). To address the inconsistent temperature in cell growth and iturin A production, a step-wise temperature control strategy was introduced in this study. During the first 24 h of cultivation, the temperature was set at 37°C for good cell growth. Subsequently, the temperature was decreased step-wise to 28°C over the next 24 h and maintained until the end of fermentation, favoring the production of iturin A. The result of batch fermentation was shown in Figure 6. The cell density in broth increased rapidly during the first 24 h and slow down as temperature decreased (24–48 h), reaching its highest value at 48 h (249.3 ± 8.5 × 10^8^ CFU/mL) (Figure 6A). This value is 81.8% higher than that of batch process without step-wise temperature control strategy. Meanwhile, glycerol was also consumed at a rapid rate and nearly depleted at 72 h (Figure 6B). The highest level of iturin A was achieved (6.6 ± 0.1 g/L) at 120 h (Figure 6C). These results clearly indicated that the fermentation period was shortened and the productivity (55.1 ± 0.3 mg/L/h) was greatly improved by the step-wise temperature control strategy.

Interestingly, the step-wise temperature control strategy did not work in the suspended cell fermentation. During the culture (0–48 h), suspended cell reactor showed a faster cell growth than process without step-wise temperature control but the maximum biomass showed only a small increase (198.0 ± 8.1 × 10^8^ CFU/mL vs. 184.0 ± 7.9 × 10^8^ CFU/mL). At the same time, the consumption rate of glycerol was also increased, accompanied by the acceleration of cell growth. Glycerol was also depleted at 72 h and the cells entered decline phase prematurely in the suspended cell reactor. Although the same glycerol consumption rate was observed, the highest iturin A concentration in the suspended cell fermentation was only 4.0 ± 0.1 g/L after 196 h which is lower than process without step-wise temperature control.

## Discussion

Biofilm growth is initiated by cell attachment to a surface of carrier, followed by surface adhesion by extracellular polymer matrix and colonization (Burmolle et al., 2006; Xu et al., 2011). Generally, porous materials with rough surface and a larger surface area work well for biofilm formation. In addition, surface properties of carrier, such as surface charge, hydrophobicity, particle diameter, and density, also play an essential role in initial cell attachment (Cheng et al., 2010). Therefore, it is necessary to choose the suitable carrier for different types of biofilm reactors and microorganisms, which can increase the attachment between microorganisms and carrier. A great variety of materials, such as stainless steel, lignocellulosic materials, polymeric materials, glass ceramic material, plastic composite, have been developed and designed for various types of biofilm reactors (Chu et al., 2014; Gu et al., 2014; Ercan and Demirci 2015; Jiang et al., 2016; Mahdinia et al., 2017; Morgan-Sagastume, 2018).

In this study, six carriers with different surface properties and structure were tested. It has been observed that not all the carriers are suitable for the *Bacillus velezensis* ND strain. Of all carriers, P (spherical filter material made of polyester fiber) showed the best performance. Thick cell layer was observed on the surface of P. It has been reported that the high degree of hydrophobicity strongly enhances adhesion of microorganisms (Silva-Dias et al., 2015; Elegbeleye and Buys, 2020). An additional aspect worthy of consideration is that iturin A secreted by *Bacillus velezensis* ND have relatively high amphiphilic (Meena and Kanwar, 2015). Therefore, it is reasonable to presume that the P with high degree of hydrophobicity is suitable for adhesion of *Bacillus velezensis* ND. The other characteristics of P are the roughness and large specific surface area (3,000 m^2^/m^3^). Moreover, P has the advantages of readily available, inexpensive, and reusable. For these reasons, P was chosen to construct the biofilm reactor after the pilot trials in shaking flasks. Its reusability was also validated in the biofilm reactor (detailed data not shown). The carrier were reused for the next batch after cleaned and dried, and the yield of iturin A was almost no change (5.4 ± 0.1 g/L). Reusability of the carrier lowers the cost of biofilm fermentation, which is important for industrial use.

The biofilm reactor we have constructed consists of a stirred-tank fermentor containing exclusively suspended cells and a packing column where the biofilm is attached. In this system, suspended cells and biofilm are co-existed. The packing column allow biofilm growth on static surfaces with low surface shear forces while the stirred-tank fermentor offers aerobic growth conditions for suspended cells. In the packing column, the liquid medium recirculated from the stirred bioreactor continuously flows down the carriers as a thin film to provide maximum gas-liquid contact, and nutrients supply for biofilm. In the stirred-tank fermentor, aerobic growth conditions support rapid proliferation of suspended cells. In brief, this system is designed to provide ideal growth conditions for suspended and biofilm population simultaneously so that higher biomass could be achieved.

During the biofilm fermentation, cell absorption to carrier was effective, and biofilm on the carrier was observed in 36–48 h. Maximum specific growth rate of both reactor were nearly the same. The total amount of biomass (in broth and biofilm) in biofilm reactor is considerably higher than suspended cell reactor (Table 1). The development of biofilm facilitated the utilization of glycerol as well as the production of iturin A. As a result, the fermentation period was shortened and volumetric productivity was greatly improved than suspended cell fermentation. Table 1 lists the important parameters of different fermentation process. The productivity and yield are most important parameters determining the economic feasibility of any bioprocess. The productivity of iturin A was increased by 66.7% and 63.3% in batch and fed-batch biofilm fermentation, respectively. The yield (Y_P/S_ and Y_X/S_) in biofilm reactor also showed obvious increase, which indicated that biofilm reactor has more effective carbon source (glycerol) utilization than suspended cell reactor. However, suspended cell reactor achieved higher Y_P/X_ value than biofilm reactor, which implied that cells that were involved in iturin A production were only a fraction of the total cells in biofilm reactor. Usually biofilms contain multiple layers of cells. In order for the cells to be active and be taking part in the reaction, nutrients must diffuse to the inner layers of cells (Qureshi et al., 2005). Therefore, it is possible that he innermost layers of biofilm have no sufficient nutrients or oxygen and did not take part in the reaction. In order to increase substrates diffusion, further optimization for medium components and flow rate of fermentation broth is necessary.

The other advantage of biofilm reactor over suspended cell reactors is the operation stability, especially in repeated-batch fermentation. When medium is removed and fresh medium introduced, cells released from mature biofilms served as the seeds for the next batch. Repeated-batch fermentation has been shown to enhance the productivity of microbial fermentations, as it reduces the cost for seed culture, and omits inoculation requirements between batches (Qu et al., 2013; Jiang et al., 2016). The advantage have the potential to lead to significant savings in terms of both time and labour (Germec et al., 2015). As shown in this study, repeated-batch fermentation in the biofilm reactor was continuous, efficient, and stable, which is important for cost-saving and achieving industrial-scale production of iturin A.

Temperature is an important factor in fermentation. Each microbial species have their optimal growth temperature while the optimal temperature for the production of secondary metabolites may vary. In addition, temperature have an effect on the rheological properties of fermentation broth and biofilm formation. Temperatures at the high end of a culture’s growth range increase the rate of cell growth, EPS production, and surface adhesion, all of which enhance biofilm formation (Di Ciccio et al., 2015). Our results showed that higher temperature during the early time of fermentation lead to faster cell growth and the maximum specific growth rate (μ_max_) reached 0.4 h^−1^. The cell dry weight in both broth and biofilm increased and the fermentation period was shortened under the step-wise temperature control strategy. As a result, the productivity and yield (Y_P/S,_ Y_X/S_ and Y_P/X_) were improved significantly (Table 1).

However, it is worth noting that the step-wise temperature control strategy did not work in the suspended cell reactor. The rate of cell growth and glycerol consumption increased but iturin A production decreased in the suspended cell fermentation. Biofilms are well known for their long-term activity and enhanced tolerance to toxic substances and other adverse conditions compared to suspended cells (Rosche et al., 2009). Therefore, this might be explained as follows: higher temperature result in faster growth and increased biomass so that the cells entered decline phase prematurely in the suspended cell reactor, while biofilm formation contribute to the maintenance of cell viability in the biofilm reactor. As a result, the step-wise temperature control strategy led to a 131.9% increase of iturin A productivity in batch biofilm fermentation than suspended cell fermentation.

No comparable value of iturin A production in biofilm reactor has been published in the literature. The parameters of iturin A was compared with other lipopeptides production in biofilm bioreactors (Table 3). The obtained iturin A productivity and yield (Y_P/S_ and Y_P/X_) in this study was higher than that reported for rotating discs bioreactor and bubbleless membrane reactor. This may be attributed to better developed biofilm which resulted the higher biomass ratio of biofilm vs planktonic cells (0.64) in this study. Actually, the biomass ratio of biofilm vs planktonic cells in the rotating discs bioreactor and bubbleless membrane reactor was 0.12 and 0.04, respectively, which indicated that most of the cells were planktonic cells (ratio was calculated based on the data given in the references). The biomass ratio of biofilm vs planktonic cells in the biofilm reactor reported by Brück et al. reached 2.1 and this could be one reason for its relatively higher surfactin productivity and yield. However, it should be mentioned that the productivity and yield are affected by many factor, such as strain, substrate, oxygen supply and other conditions during fermentation process.

**TABLE 3:** Comparison of parameters for production of lipopeptides in bioreactor.

Product	Productivity (mg/L/h)	Y_X/S_ (g/g)	Y_P/S_ (mg/g)	Y_P/X_ (mg/g)	μ_max_ (/h)	Time (h)	Type of reactor	References
Iturin A	55.1	0.13	74.8	577.8	0.40	120	Biofilm reactor	This study
Total lipopeptides^b^	14.9^a^	0.21	71.5	337	—	72	Rotating discs bioreactor	Chtioui et al. (2014)
Total lipopeptides^b^	20.2^a^	0.21^a^	36.3^a^	172.7^a^	—	72	Membrane bioreactor	Coutte et al. (2010)
Surfactin	195.2^a^	0.20	87.4^a^	437.1^a^	0.38	45	Biofilm Bioreactor	Brück et al. (2020)
Surfactin	4.9^a^	0.31	11.7^a^	37.9	—	72	Biofilm reactor	Zune et al. (2014)

^a^ Values are calculated based on the data given in the listed references.

^b^ Total lipopeptides of surfactin and fengycin.

In conclusion, a novel two-compartment biofilm reactor is designed and constructed. Polyester fiber with sphere shape was chosen as the carrier of biofilm because of having high degree of hydrophobicity and rough surfaces. In this biofilm reactor, higher biomass could be achieved because of ideal growth conditions for suspended cell and biofilm population simultaneously. The fermentation period was shortened and volumetric productivity was greatly improved than suspended cell fermentation. Compared to suspended cell fermentations, the productivity of iturin A in batch and fed-batch biofilm fermentation were increased by 66.7% and 63.3%, respectively. The step-wise temperature control strategy resulted in 131.9% increase of iturin A productivity compared to suspended cell fermentation.

## Data Availability

The original contributions presented in the study are included in the article/Supplementary Material, further inquiries can be directed to the corresponding author.
