# Supplementary material for: Enhanced iturin a production in a two-compartment biofilm reactor by Bacillus velezensis ND
Source: Front Bioeng Biotechnol. 2023 Jan 19;11:1102786. doi: 10.3389/fbioe.2023.1102786 (PMC9893019; doi:10.3389/fbioe.2023.1102786)

**Supplementary Fig. 1** HPLC results of the iturin A standard and fermented sample.

Calculate the concentration of iturin A by accumulating the peak areas of 1, 2 and 3 in the graph.


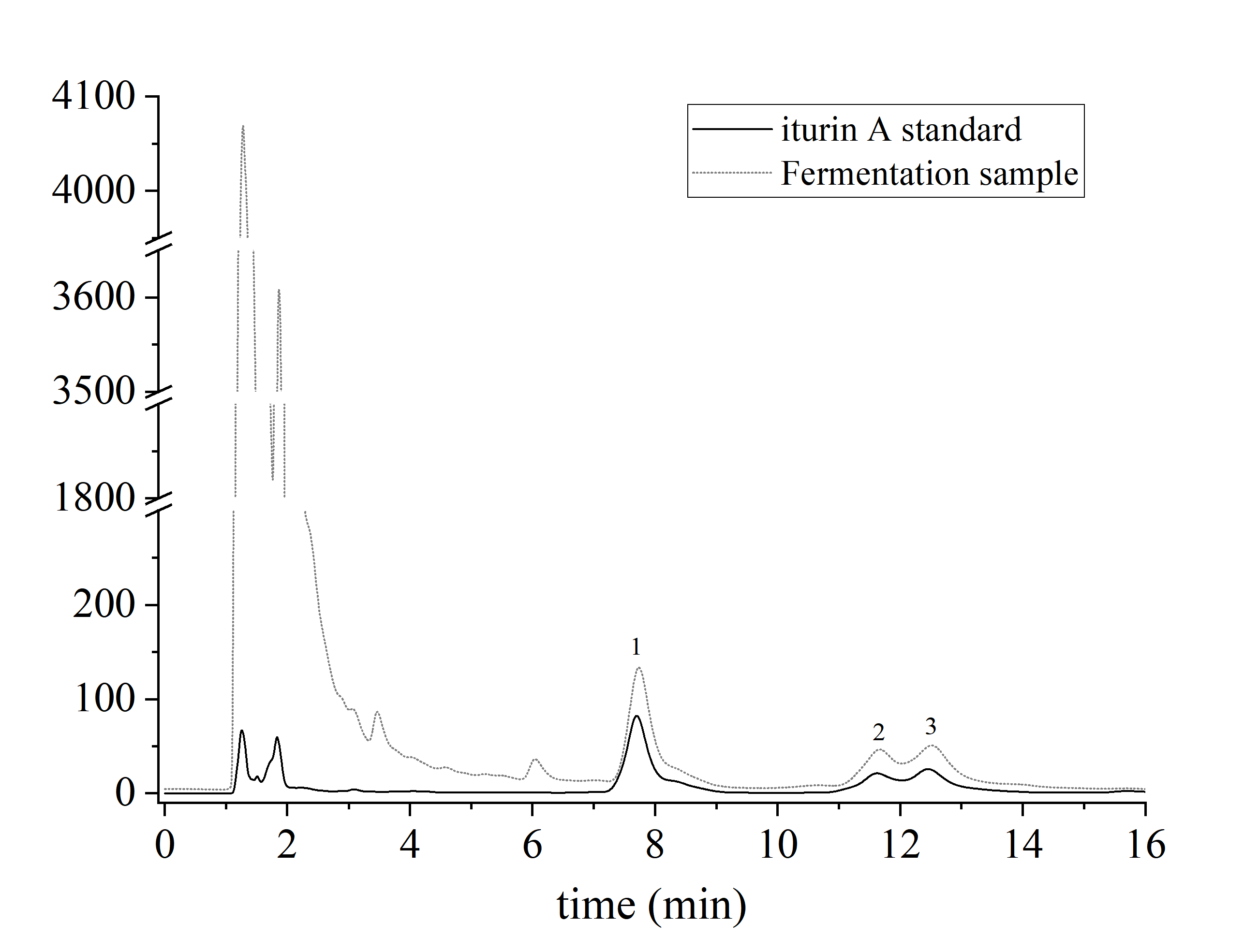

Supplement: Supplementary file 1 [file DataSheet1.docx]
